# Squalene Emulsion Manufacturing Process Scale-Up for Enhanced Global Pandemic Response

**DOI:** 10.3390/ph13080168

**Published:** 2020-07-28

**Authors:** Tony Phan, Christian Devine, Erik D. Laursen, Adrian Simpson, Aaron Kahn, Amit P. Khandhar, Steven Mesite, Brad Besse, Ken J. Mabery, Elizabeth I. Flanagan, Christopher B. Fox

**Affiliations:** 1 Infectious Disease Research Institute, 1616 Eastlake Avenue E #400, Seattle, WA 98102, USA; lphan@agcbio.com (T.P.); chris.devine@idri.org (C.D.); erik.laursen@idri.org (E.D.L.); adriansimpson@verily.com (A.S.); aaron.kahn@hotmail.com (A.K.); amit.khandhar@pailifesciences.com (A.P.K.); 2 Microfluidics Corp., 90 Glacier Drive #1000, Westwood, MA 02090, USA; SMesite@idexcorp.com (S.M.); BBesse@idexcorp.com (B.B.); 3 Pall Corp., 20 Walkup Drive, Westborough, MA 01581, USA; ken_mabery@pall.com; 4 Sartorius Stedim North America Inc., 565 Johnson Avenue, Bohemia, NY 11716, USA; Elizabeth.Flanagan@sartorius.com; 5 Department of Global Health, University of Washington, Seattle, WA 98102, USA

**Keywords:** squalene emulsion, vaccine adjuvant, nanoemulsion, process scale-up, emulsion manufacturing, adjuvant manufacturing, pandemic response

## Abstract

Squalene emulsions are among the most widely employed vaccine adjuvant formulations. Among the demonstrated benefits of squalene emulsions is the ability to enable vaccine antigen dose sparing, an important consideration for pandemic response. In order to increase pandemic response capabilities, it is desirable to scale up adjuvant manufacturing processes. We describe innovative process enhancements that enabled the scale-up of bulk stable squalene emulsion (SE) manufacturing capacity from a 3000- to 5,000,000-dose batch size. Manufacture of concentrated bulk along with the accompanying viscosity change in the continuous phase resulted in a ≥25-fold process efficiency enhancement. Process streamlining and implementation of single-use biocontainers resulted in reduced space requirements, fewer unit operations, and minimization of cleaning requirements. Emulsion physicochemical characteristics were measured by dynamic light scattering, laser diffraction, and HPLC with charged aerosol detection. The newly developed full-scale process was demonstrated by producing two 5,000,000-dose batches of bulk concentrated SE. A scale-up of adjuvant manufacturing capacity through process innovation enables more efficient production capabilities for pandemic response.

## 1. Introduction

Squalene emulsions (SE) represent an important component of pandemic preparedness. An estimated 200M doses of vaccines containing squalene-based oil-in-water (*o/w*) emulsions have been administered to humans, making them the most widely used class of adjuvant formulations, except for aluminum salts [1]. Squalene-based emulsions have been shown to help potentiate immune responses against a variety of diseases in preclinical and clinical testing, such as malaria, tuberculosis, leishmaniasis, meningitis, etc. [2], and various squalene-based formulations (e.g., MF59^®^, AS03™, and AF03™) have been developed by large pharmaceutical companies and included in licensed influenza vaccine products. Pandemic vaccines, in particular, can benefit from the antigen dose sparing effects of squalene emulsions. Indeed, since vaccine production capacity is currently inadequate to meet the demand of a global pandemic, squalene emulsions may play an essential role in expanding vaccine coverage [3]. For example, GSK is making available their AS03 adjuvant for COVID-19 vaccine development partners [4].

While some governments maintain stockpiles of squalene-based emulsions for pandemic preparedness [5], additional adjuvant manufacturing capacity is needed to achieve global pandemic response capabilities [6]. In particular, developing countries with vaccine manufacturing programs could benefit from the increased availability of adjuvants. Previous technology transfer efforts have sought to establish local adjuvant manufacturing capacity [7]. Nevertheless, such efforts have been limited to a relatively small manufacturing scale.

The Infectious Disease Research Institute (IDRI) has developed a squalene-phospholipid stable emulsion (SE) that has moved into clinical testing with influenza vaccines, demonstrating dose-sparing capacity [8]. The composition of SE differs from that of other commercially produced squalene emulsions, with SE being the only clinical stage squalene emulsion to employ phospholipid as the primary emulsifier [1]. In the present report, we describe the scale-up of bulk SE adjuvant manufacturing from 3000 to 5,000,000 doses/batch, including the development of innovative process efficiency improvements to allow for a concentrated bulk emulsion manufacturing capacity even in a relatively small Current Good Manufacturing Practice (cGMP) facility.

## 2. Results and Discussion

Scale-up objectives. The pre-determined goal of the project was to establish the capability to manufacture 50 million total doses of squalene emulsion adjuvant within 3 months of a pandemic declaration. IDRI’s cGMP facility is a small 4000 ft^2^ facility staffed by a team that works a single shift. Assuming some days may be needed for equipment maintenance, we estimated that 50 working days in a 3-month period could be dedicated to adjuvant manufacturing, translating into a 1M dose daily batch size required to meet the pre-determined objective. Due to the facility space constraints, manufacturing multiple batches in parallel lines was not a feasible alternative. To support Phase 1/2 clinical trials, SE had generally been manufactured at the 1-L scale, which is equivalent to ~3000 doses after accounting for manufacturing losses and assuming 250 µL for a human dose (SE is typically manufactured at 4% *v/v* oil which is designed for bedside mixing with vaccine antigen at 1:1 *v:v* ratio for a final injection composition of 2% *v/v* oil, see Table 1). Thus, the pre-determined objective required scaling from 3000 doses to 1M doses per day, a ~300-fold increase in scale (i.e., from 1-L batch size to 300-L batch size). Performance criteria to assess success of manufactured emulsions were as follows: particle diameter (Z-ave) of ≤100 nm as measured by dynamic light scattering, oil and excipient content within 20% of target values as measured by HPLC with charged aerosol and diode array detection, and a homogeneous milky-white visual appearance.

**Figure 1:**
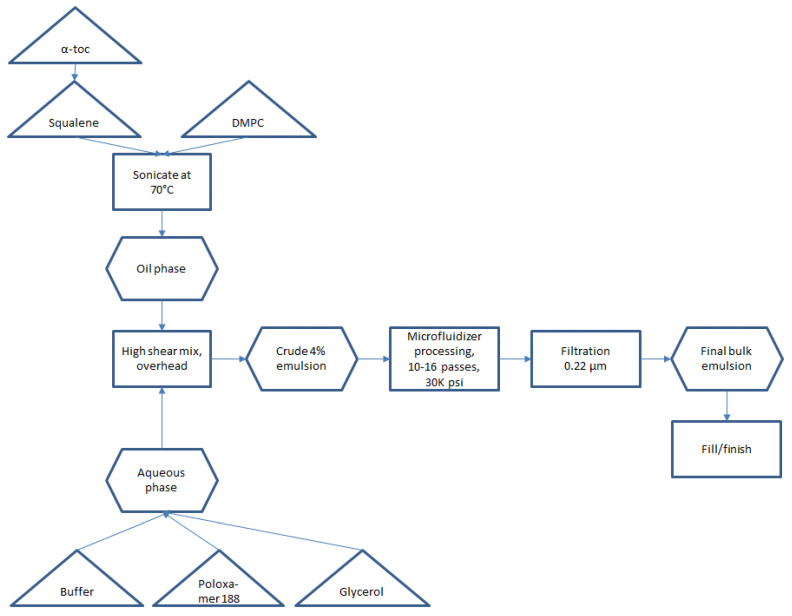
Small-scale (≤1 L) process flow diagram for production of SE.

Small scale manufacturing process. The basic manufacturing process and composition for small scale SE (1-L batch) was reported by Fox et al. [9]. Since 2013, some changes in the reported SE composition have been made, namely replacing egg phosphatidylcholine with synthetic DMPC [10] and the addition of a small amount of α-tocopherol as an antioxidant (Table 1) [11]. The resulting small scale process is shown in Figure 1. Some inefficient or difficult-to-scale steps can be noted in Figure 1, namely water bath sonication to disperse components (difficult to scale), separate containers required for oil and aqueous phases (space contraints in cGMP suite for multiple large containers), and a high number of passes required on the Microfluidizer processor (at an anticipated flow rate of ~3 L/min, a 300-L batch size would require 27 h to process 16 passes). Based on these calculations, it was decided to develop innovations to improve process efficiency and reduce space or equipment requirements prior to a full scale-up.

Removal of heated sonication step. To enable process simplification and scale-up feasibility, we evaluated the impact of removing the elevated temperature sonication step. Sonication in a heated water bath was originally presumed to be necessary to efficiently disperse the emulsifier (DMPC) in the oil phase to promote more rapid particle size reduction in subsequent processing steps. However, elimination of the heated sonication step appeared to have no detrimental effect on the resulting physical characteristics of the manufactured emulsions (Table 2). Therefore, subsequent emulsion process development omitted the heated sonication step.

**Figure 2:**
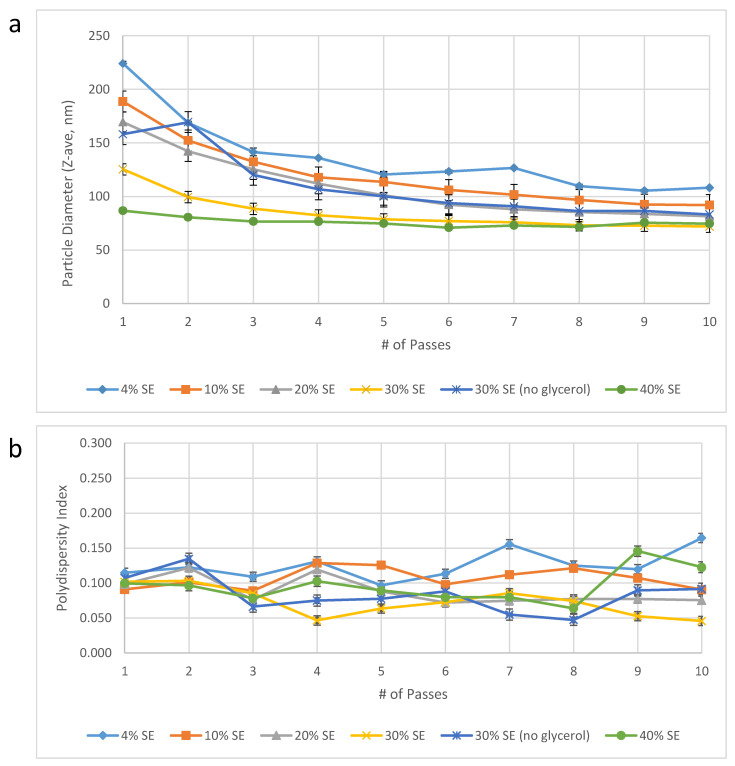
Emulsion droplet diameter (**a**) and polydispersity index (**b**) as a function of dispersed phase volume percent and number of Microfluidizer processing passes. Droplet diameter was measured within one month following manufacture. Error bars represent standard deviation of three measurements from one batch of emulsion produced at each dispersed phase content level. Note that the specific volume of DMPC was not accounted for, thus the concentration of DMPC and other aqueous phase excipients may have varied between ~1–13% from the target concentrations [13].

Dispersed phase concentration. In order to reduce the Microfluidizer processing time required, we evaluated the effect of manufacturing more concentrated emulsions that could be diluted with water post-Microfluidizer processing to the target concentration. In effect, this would reduce the volume of emulsion in the Microfluidizer processing step and thus decrease the processing time. On the other hand, we anticipated that increased oil and excipient content could potentially require additional energy input (i.e., the number of passes through the Microfluidizer processor) to achieve the target particle size. Surprisingly, we found that increasing oil and excipient concentrations resulted in a dramatic reduction in the required number of passes to achieve the target particle size (Figure 2). Thus, manufacturing a 30% *v/v* oil-in-water emulsion reduced the volume of emulsion required for Microfluidizer processing as well as the number of passes required to achieve the target particle size. Specifically, at 30% *v/v* emulsion there was a 7.5-fold reduction in volume required for Microfluidizer processing compared to 4% *v/v* oil-in-water emulsion, and only 2–3 passes were required to achieve the target particle size compared to 10–16 passes for the 4% *v/v* oil-in-water emulsion. Therefore, increasing the emulsion concentration to 30% *v/v* oil resulted in at least a 25-fold process efficiency improvement (7.5-fold volume reduction * 10/3 minimum reduction in number of passes). Notably, a higher oil concentration (40% *v/v* oil) was attempted to see if an additional benefit was possible but it was found not to be suitable due to practical processing difficulties associated with the high viscosity of the 40% emulsion.

The remarkable finding that increasing the dispersed phase (oil) content from 4% to 30% *v/v* resulted in more rapid particle size reduction during Microfluidizer processing, and a smaller particle size overall given the same number of passes through the Microfluidizer processor, may be explained due to the viscosity ratio [12]. The smallest achievable particle size is typically expected to remain unchanged or even increase with a higher oil phase content (e.g., 4% to 30%) when the oil:emulsifier ratio is held constant as it was in our experiments. However, if the viscosity ratio of the dispersed phase/continuous phase (*η_d_/η_c_*) is affected when the dispersed phase volume is increased, a smaller particle size may be achievable [12]. Thus, assuming a turbulent-viscous flow regime, the maximum particle size that can persist undisrupted (*d_max_*) can be described as follows:

(1) $$d_{max}=\frac{\gamma }{{\left(\varepsilon \eta_{c}\right)}^{\frac{1}{2}}}$$

where *γ* is the interfacial tension, *ε* is the power density, and *η_c_* is the continuous phase viscosity; meaning that *d_max_* is inversely proportional to *η_c_* [12].

The importance of the viscosity ratio with regards to particle size reduction during Microfluidizer processing is especially relevant to the emulsion composition and process described in the present work. In order to enable post-Microfluidizer processing dilution with water, we increased all excipient content in the 30% *v/v* emulsion such that dilution with water would result in the desired target excipient concentrations at 4% *v/v* emulsion. Thus, all excipients including the continuous aqueous phase excipients were increased 7.5-fold from the 4% *v/v* emulsion composition in order to manufacture the 30% *v/v* emulsion composition (Table 1). Therefore, the viscosity of the continuous aqueous phase is increased with respect to the viscosity of the dispersed oil phase, which remains constant (Table 3). In particular, glycerol contributes to the viscosity of the aqueous phase since the omission of glycerol in the aqueous phase of the 30% *v/v* emulsion composition results in no viscosity increase compared to the aqueous phase of the 4% *v/v* emulsion composition, and the 30% *v/v* emulsion made without glycerol has larger particle size than the same emulsion made with glycerol. However, the situation is even more complex due to the emulsifier DMPC, which has a substantial impact on the viscosity of both the aqueous phase and the oil phase during emulsion processing. In any case, it appears that the improved particle size reduction performance of the concentrated emulsions can be attributed, at least in part, to increased viscosity of the continuous aqueous phase compared to the dispersed oil phase.

One-pot mixing. To evaluate the potential to eliminate the need for separate containers for the oil and aqueous phases, we manufactured emulsions using the one-pot approach to compare to emulsions made with the traditional separate container approach. We found that emulsions that had been prepared with oil and aqueous phases in a single container (one-pot approach) demonstrated equivalent physicochemical properties to emulsions manufactured in the traditional manner (separate containers for oil and aqueous phases) (Table 4). The one-pot mixing approach further enhanced efficiency and reduced space and equipment requirements.

**Figure 3:**
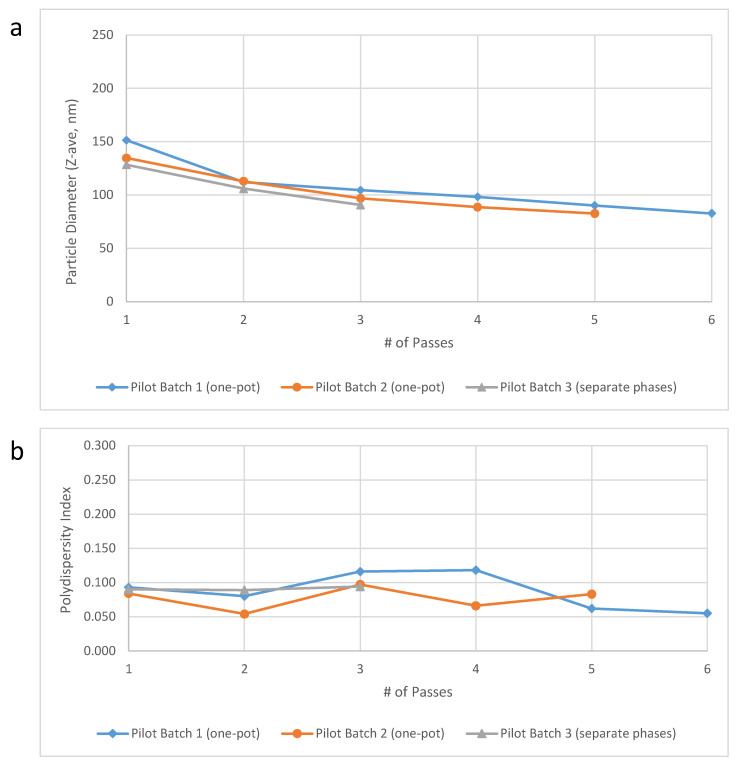
Droplet diameter (**a**) and polydispersity index (**b**) as a function of number of passes through the Microfluidizer processor in 30% *v/v* oil-in-water emulsion batches produced at the pilot scale (10 L) using the one-pot approach or the traditional separate phases approach as indicated.

**Figure 4:**
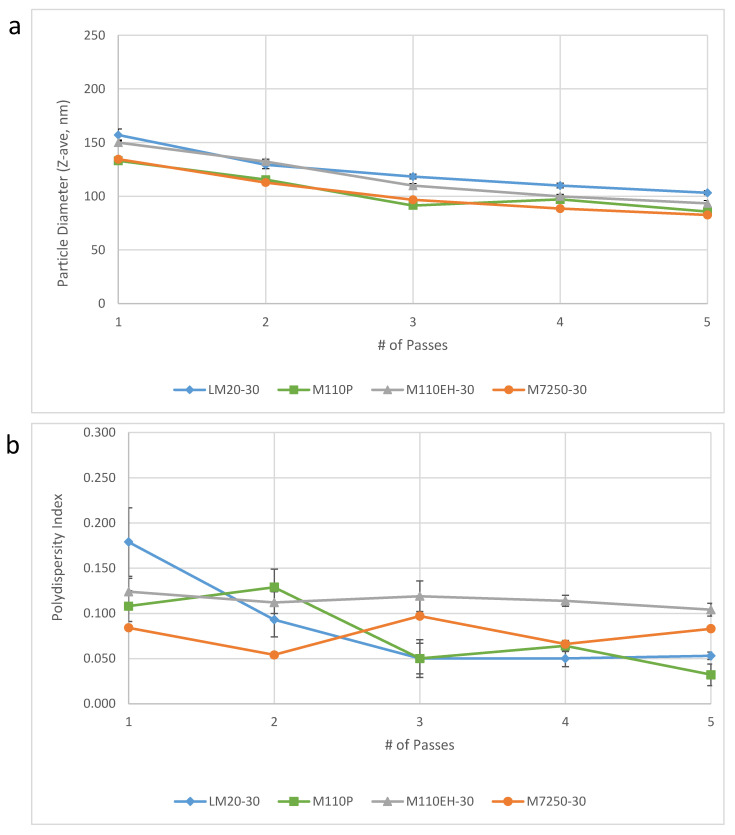
Droplet diameter (**a**) and polydispersity index (**b**) as a function of number of passes through the indicated Microfluidizer processor model. Error bars represent the standard deviation from three measurements from the same aliquot. The M-7250-30 data are taken from Figure 3. Batch size produced for each model was 10 L (M7250-30), 300 mL (M110-EH), 200 mL (M110P), and 100 mL (LM20). Typical representative flow rate for each Microfluidizer model when processing at 30,000 psi is 2.8 L/min (M7250-30), 400 mL/min (M110-EH), 110 mL/min (M110P), and 80 mL/min (LM20). Prior to Microfluidizer processing, high shear mixing conditions and equipment varied somewhat for the different batches although minimal impact is anticipated from these differences.

Pilot scale testing. Pilot scale-up efforts were performed at the 10-L scale in the Microfluidics testing laboratory using the M7250-30 model Microfluidizer processor. Verification of acceptable performance at this scale using the one-pot approach compared to separate oil and aqueous phases was achieved for key physical and chemical properties of interest (Table 5). Furthermore, the target particle size was obtained after only 3–4 passes (Figure 3). With an anticipated flow rate of ~3 L/min and only three required passes, the M7250-30 model would enable processing of 50 L of 30% SE (1.2 M doses) in <1 h, a dramatic improvement to the 27 h initially estimated (see above). However, partial or complete plugging of the M7250-30 Interaction Chamber^TM^ due to DMPC aggregates was experienced for all three pilot scale batches despite the use of a high shear mixer, and additional mitigation was recommended in future to reduce the risk of plugging. As a result, we implemented milling of the DMPC powder prior to the formulation of subsequent large-scale batches (see below). Additional testing of squalene emulsions in the Microfluidics testing laboratory resulted in the identification of optimized equipment parameters including Interaction Chamber selection, constant pressure profile preferable to synchronous pressure profile, discrete passes preferable to recirculating continuous passes, and addition of 100 psi backpressure preferable to no backpressure, consistent with a previous report [14]. Notably, the results obtained on the large scale M7250-30 model demonstrated comparable performance to three consecutive batches of emulsion made on smaller Microfluidizer models that were used for small scale production at IDRI (Figure 4).

Filtration development. The 30% *v/v* squalene emulsion presented a significant filtration challenge due to the high oil content. The pilot scale emulsions produced as described above were employed to evaluate filterability using a variety of filters. Emulsions were passed through 47 mm filter discs at constant flow until the pressure reached 30 psi, after which the filter capacity was calculated (Table 6). In general, polyethersulfone (PES) membrane material was found to achieve higher filtration capacity than cellulose acetate or polyvinylidene fluoride (PVDF) membranes. Moreover, the highest filtration capacities were achieved by using dual layer filters that employed a larger pore size (0.35–0.8 µm) followed by a 0.2 µm final filter membrane. Based on these data, dual layer PES filters in large capsules (20”–30”) were recommended for full-scale filtration (see below). Additional bacterial retention testing by the selected filter vendor resulted in a recommendation for employing two serial 30” filter capsules to minimize risk, although this approach was not evaluated in the present report.

Closed system with single-use containers and inline mixing. Single-use containers in a largely closed system configuration were implemented in preparation for large scale manufacture to reduce cleaning requirements and the risk of product contamination. Thus, the emulsion components were added to a single-use bag mixing system with a low shear mixing impeller. After component addition, the next step prior to Microfluidizer processing was high shear mixing to generate a crude emulsion (see Figure 1). To further reduce space requirements as well as exposure to the environment, we evaluated the feasibility of replacing open system overhead high shear mixing with closed system inline high shear mixing. Laser diffraction particle size analysis revealed comparable performance in particle size reduction of the inline mixer compared to the overhead mixer depending on processing time, with either method producing median particle diameters (Dv50) of ~3–7 µm.

Revised Process and Full-Scale Production. In order to meet the pre-established target production capacity of 1M doses/day, a batch size of 50 L of 30% *v/v* SE would be sufficient. However, based on the remarkable process efficiency improvements described above, it was determined that target production capacity could be increased to 5 M doses/day (200 L of 30% SE). Thus, 200-L single-use mixing bags and associated equipment and materials were acquired. Nevertheless, initial large-scale testing was carried out at the 50-L scale to identify challenges to be addressed prior to scaling to 200 L. At the 50-L scale, some practical difficulties were encountered. For example, the backpressure applied to the Microfluidizer did not achieve the target pressure, which resulted in five discrete passes required to achieve a particle size <100 nm and filtration issues due to reaching the maximum pressure that the silicon tubing was rated for (20 psi) prior to filtration of the entire batch. In order to address these challenges, modifications to the order and method of component addition were implemented, the Microfluidizer processor’s backpressure valve was adjusted, and thermoplastic elastomer tubing rated for up to 43 psi (PharMed^®^ BPT) was acquired prior to production at the 200-L scale.

Two separate batches were manufactured at the full 200-L scale. Raw materials were prepared prior to emulsion production by milling the DMPC and kitting all powders (DMPC, poloxamer 188, buffer salts). For accurate addition of α-tocopherol, it was first added to a 4-L volume of squalene in a 5-L container using a benchtop scale and mixed using a stir bar. This squalene/α-tocopherol mixture was then added to the remaining squalene in the 200-L mixing unit using a peristaltic pump while the impeller was rotating. Following sequential addition of all other emulsion components, the crude 200-L mixture was then processed through the high shear inline mixing unit for approximately four recirculating passes, achieving a median particle diameter (Dv50) of 4.0 µm (Table 7). The crude emulsion was then processed for three discrete passes on the Microfluidizer processor (M7250-30), resulting in a particle size of 94 ± 2 nm. The processed emulsion was pumped through a 0.45/0.2 PES filter capsule using a peristaltic pump. The entire batch was filtered using a single 30” filter capsule within 70 min, with 23 psi the maximum pressure achieved during filtration at a flow rate of 3.3 L/min. The final yield was 195 L of 30% *v/v* emulsion. See Table 7 for a summary of physicochemical characterization results.

**Figure 5:**
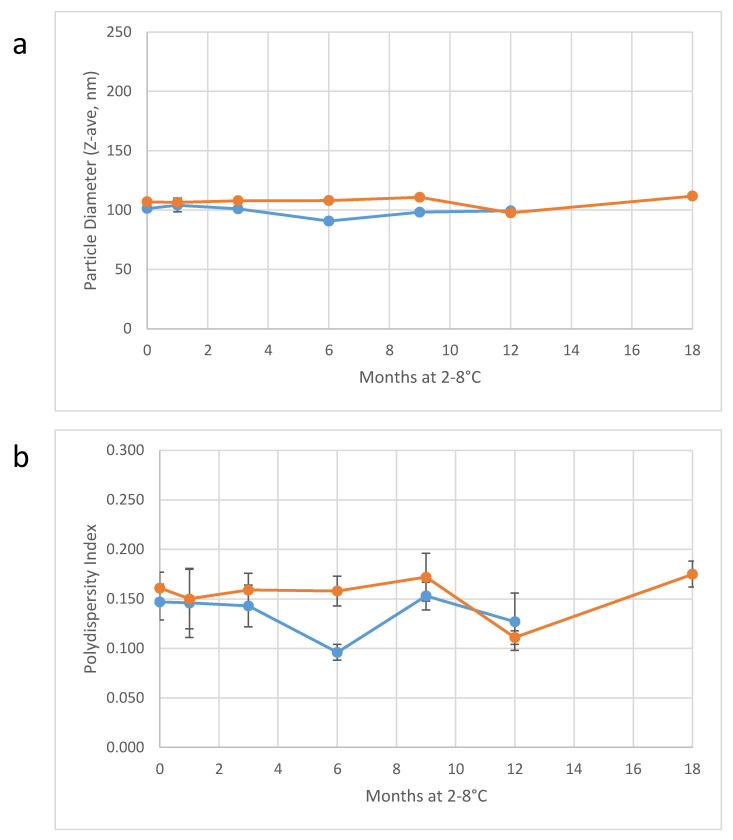
Physical stability of the two large-scale batches from Table 7 when stored at 2–8 °C. Droplet diameter (**a**) and polydispersity index (**b**) remain stable for a minimum of 12–18 months. Error bars represent the standard deviation of 8–9 measurements.

The second 200-L batch was produced in a similar manner to the first batch with minor practical adjustment in the order of component addition, which proceeded sequentially as follows: squalene/α-tocopherol as described above, water-for-injection (WFI), buffer salts, poloxamer 188, DMPC, and glycerol. Median particle diameter after four recirculating high shear mixing passes was 4.2 µm, and particle diameter following three discrete passes through the Microfluidizer processor was 92 ± 2 nm (Table 7). Filtration was conducted at a higher flow rate (4.9 L/min), allowing completion of filtration within 45 min at a maximum pressure of 21 psi and a final yield of 196 L of 30% *v/v* emulsion. All characterization results were within expected ranges for the second 200-L batch (Table 7). Importantly, particle diameter monitoring of the two large-scale batches of 30% *v/v* SE demonstrates long-term physical stability of the concentrated emulsions (Figure 5). Thus, the 30% *v/v* emulsion can be held for an extended period of time, if necessary, prior to dilution and fill/finish into vials at the target concentration.

**Figure 6:**
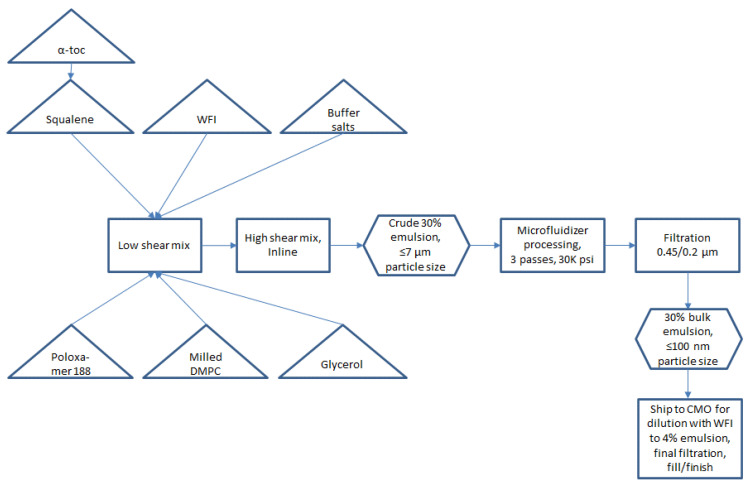
Large-scale (200 L) process flow diagram for one-pot production of SE.

The optimized process flow diagram for production of bulk concentrated squalene emulsion, incorporating the process innovations described above and demonstrated successfully at the 200-L scale (for 5M doses), is shown in Figure 6. The process represents a more streamlined approach compared to the original process (Figure 1). There is no need for sonication or for separate large containers for the aqueous phase and oil phase. Overhead high shear mixing has been replaced by inline high shear mixing, and Microfluidizer processing time has been dramatically reduced by increasing dispersed phase content (and thus reducing total processed emulsion volume). The process is performed using single-use disposable units and can be completed within a normal day shift, with raw materials and equipment prepared for use ahead of time.

Despite the successful scale-up demonstration of squalene emulsion production presented here, some gaps remain to be addressed. For instance, potency data should be obtained to confirm the new process does not impact emulsion bioactivity. Moreover, the present report describes bulk emulsion production, but matching fill/finish capacity is also needed to provide the final drug product for distribution. Furthermore, evaluation of the optimal container and closure composition, and whether there is an added benefit of inert gas overlay, for long-term storage of 30% *v/v* SE is needed to maximize value. In addition, filter challenge and validation studies are required to minimize the risk of bacterial contamination. Other areas that merit more effort include the identification and scale-up of alternative sustainable sources of raw squalene to replace the current source (shark liver) [15,16,17,18], the development and implementation of thermostable presentations of squalene emulsions, such as dried formulations [19,20], and additional efforts to build local capacity for squalene emulsion production in developing countries [7,9,11,21]. Research and development in each of these areas is ongoing in our laboratory and elsewhere.

## 3. Materials and Methods

Materials. 1,2-dimyristoyl-*sn*-glycero-3-phosphocholine (DMPC) was purchased from Lipoid LLC (Newark, NJ, USA), Avanti Polar Lipids (Alabaster, AL, USA), or NOF America (San Mateo, CA, USA). Squalene was purchased from Sigma-Aldrich (St. Louis, MO, USA) and SEPPIC (Fairfield, NJ, USA). α-tocopherol and poloxamer 188 were purchased from Spectrum Chemical (Gardena, CA, USA). Glycerol was obtained from Fisher Chemical (Waltham, MA, USA) or Spectrum Chemical. Monobasic and dibasic ammonium phosphate were purchased from J.T. Baker (San Francisco, CA, USA). Water was obtained from in-house RO/DI system, or from in-house Milli-Q (Millipore) system. Filters were obtained from Pall (Port Washington, NY, USA) and Sartorius (Bohemia, NY, USA). Single-use mixing bags, process bags, powder handling bags, shipping bags, and liners were also purchased from Pall.

Process Equipment. Microfluidizer^®^ models M7250-30, M110EH-30, M110P, and LM20-30 from Microfluidics (Westwood, MA, USA) were employed [22]. High shear mixers included the ZC0 in-line mixer from Quadro (Waterloo, ON, USA), the L4RT and BX60 overhead mixers from Silverson (East Longmeadow, MA, USA), and the T50 rotostator mixer from IKA (Wilmingon, NC, USA). The Comil U5 powder milling unit was obtained from Quadro. The 200-L Allegro mixer, 200-L Allegro plastic collapsible totes, and 200-L Allegro stainless steel transportation tote were purchased from Pall. An IFS4 model flatbed floor scale was obtained from Sartorius and a top loading benchtop balance was obtained from Mettler Toledo (Columbus, OH, USA). A peristaltic pump was purchased from Flexicon (Bethlehem, PA, USA).

Analytical characterization. Emulsion particle size and polydispersity index was determined via dynamic light scattering with the Malvern Instruments (Worcestershire, UK) Zetasizer Nano-S, -ZS, or –APS, or via laser diffraction using the Horiba Scientific (Irvine, CA, USA) LA-950/960 or the Beckman Coulter (Brea, CA, USA) LS230. Particle sizing sample preparation involved diluting emulsions into water. HPLC with charged aerosol detection was employed to quantify squalene and DMPC content. Sample preparation for HPLC involved combining 10 μL of emulsion with 990 μL mobile phase B (1:1 [v:v] methanol:chloroform with 20 mM ammonium acetate and 1% acetic acid) in glass HPLC vials. A volume of 5 µL of sample was then injected on to a Waters Co. (Milford, MA, USA) XBridge C18 column at 30 °C on an Agilent Model 1100 HPLC (Santa Clara, CA, USA). A 1 mL/min flow gradient consisting of mobile phase A (75:15:10 [v:v:v] methanol:chloroform:water with 20 mM ammonium acetate and 1% acetic acid) and mobile phase B was employed over 18 min (80:20 A:B at 0 min, 100:0 A:B at 9 min, 50:50 A:B at 13 min, and 80:20 A:B at 18 min). Detection was performed by an ESA Biosciences (Chelmsford, MA, USA) Corona Charged Aerosol Detector (CAD) or a ThermoFisher Scientific (Waltham, MA, USA) Corona Veo CAD. Quantitation was performed using standards of each component prepared in mobile phase B and injected at different volumes. Osmolality was measured by freezing point depression using an Advanced Instruments (Norwood, MA, USA) Osmometer Model 2020, pH and conductivity were measured using standard meters, and viscosity was measured using a Brookfield DV-E digital viscometer (Middleboro, MA, USA).

## 4. Conclusions

By re-engineering the SE production process, we have enabled a ≥25-fold increase in efficiency while also minimizing required space, container, and cleaning requirements. Using the revised production process, we have demonstrated the capability to produce a 200-L batch size of concentrated bulk squalene emulsion, equivalent to 5,000,000 doses, in a small manufacturing facility in one day. Improved adjuvant manufacturing approaches such as the one described here could play an important role in pandemic response by enabling vaccine dose sparing.

## Figures and Tables

**Table 1:** Squalene emulsion (SE) composition.

Component	Concentration in 4% *v/v* Oil SE (mg/mL)	Concentration in 30% *v/v* Oil SE (mg/mL)
**squalene**	34	257
**α-tocopherol**	0.2	1.5
**DMPC**	7.6	57
**poloxamer 188**	0.36	2.7
**glycerol**	22.7	170
**ammonium phosphate, monobasic**	2.7	20.5
**ammonium phosphate, dibasic**	0.17	1.2
**water-for-injection (WFI)**	QS	QS

**Table 2:** Effect of sonication on emulsion droplet characteristics.

Name	Number of Emulsions Manufactured	Particle Diameter(Z-ave, nm)	Polydispersity Index (PdI)
**Sonication at ~70 °C**	4	79.6 ± 7.1	0.047 ± 0.004
**No sonication or heating**	4	86.1 ± 2.6	0.045 ± 0.005

Note: Emulsions in this experiment were manufactured at 100–200-mL batch size, 4% *v/v* squalene concentration and 10–12 recirculating passes on the Microfluidics 110-P. Differences in Z-ave and PdI were not statistically significant by Student’s *t*-test.

**Table 3:** Viscosity of oil and aqueous phases and corresponding emulsion particle size.

Description *	Viscosity (cP) **	Emulsion Particle Diameter (Z-Ave, nm) after 3 Passes (see Figure 2a)
Oil phase	14.8	N/A
Aqueous phase for 4% SE	1.4	141.5 ± 3.6
Aqueous phase for 10% SE	1.3	132.4 ± 2.1
Aqueous phase for 20% SE	1.7	125.4 ± 1.4
Aqueous phase for 30% SE	2.3	88.5 ± 1.5
Aqueous phase for 30% SE (no glycerol)	1.4	120.2 ± 0.9
Aqueous phase for 40% SE	3.9	76.6 ± 0.4

* The specific volume of DMPC was not accounted for, thus the concentration of DMPC and other aqueous phase excipients may have varied between ~1–13% from the target concentrations [13]. ** Viscosity was measured prior to addition of DMPC; DMPC impacts viscosity of both oil and aqueous phases.

**Table 4:** Effect of the one-pot approach on emulsion droplet characteristics at small scale (≤200 mL).

Name	Number of Emulsions Manufactured	Particle Diameter (Z-ave, nm)	Polydispersity Index (PdI)	pH	Conductivity (mS)
**Separate oil and aqueous phases**	3	85.6 ± 8.8	0.061 ± 0.008	5.2 ± 0.1	5.4 ± 0.1
**One-pot approach**	2	95.8 ± 18.5	0.062 ± 0.016	5.3 ± 0.1	5.7 ± 0.2

Note: Emulsions in this experiment were manufactured at 100–200-mL batch size, 30% *v/v* squalene concentration and 5 recirculating passes or 4 discrete passes on the Microfluidics 110-P. Differences in Z-ave, PdI, pH, and conductivity were not statistically significant by Student’s t-test.

**Table 5:** Pilot scale (10 L) production of 30% *v/v* SE using one-pot or separate phase methods.

Name	Number of Emulsions Manufactured	Particle Diameter (Z-ave, nm)	Polydispersity Index (PdI)	% Target Squalene Conc.	% TargetDMPC Conc.
**One-pot**	1	96.8	0.097	98 ± 2	103 ± 2
**Separate phases**	1	90.6	0.094	94 ± 5	96 ± 7

Note: Emulsions in this experiment were manufactured at 10-L batch size, 30% *v/v* squalene concentration on the Microfluidics M-7250-30 with a recirculating chiller set to 5 °C. Particle diameter values were measured immediately after the 3rd discrete pass for each batch. The final number of discrete passes was 5 and 3, respectively, for the one-pot and separate phase batches. HPLC measurements were collected 26 mon after manufacture with emulsions stored at 2–8 °C, and the standard deviation from three measurements is shown.

**Table 6:** Evaluation of filter capacity regarding 30% *v/v* SE.

Filter Pore Size (µm)	Vendor *	Filter Membrane Material	Flux Tested (LMH)	Calculated Capacity (L/m^2^)
0.35/0.2	Sartorius	PES	192	93
0.45/0.2	Sartorius	PES	175	66
0.45/0.2	Sartorius	PES, high bubble point	169	55
0.45/0.2	Sartorius	PES, surface modified	175	46
0.45/0.2	Sartorius	Cellulose acetate	157	40
0.8/0.2	Sartorius	PES	174	85
0.45/0.2	Pall	PES	545	57
0.65/0.2	Pall	PES	545	46
0.2/0.2	Pall	PES/PVDF	545	23
0.2/0.2	Pall	PVDF	545	2

* Sartorius filters were tested the day after emulsion production; Pall filters were tested 2 weeks following production.

**Table 7:** Large-scale (200 L) 30% *v/v* SE batch characterization.

	Target Range	200-L Batch A	200-L Batch B
Date of Manufacture	For information only	22 May 2018	28 November 2018
Particle diameter after 4 recirculating passes on high shear mixer (D50; μm)	≤7.0	4.0	4.2
Particle diameter after 3 discrete Microfluidizer passes (Z-ave; nm)	≤100	94 ± 2	92 ± 2
Yield % (filtered volume)	For information only	96% (192 L)	97% (193 L)
Squalene content (% of target)	80–120	99 ± 5	114 ± 7
DMPC content (% of target)	80–120	103 ± 5	119 ± 7
pH	5.0–5.5	5.2	5.2
Processing time (h)	≤10	7	7
